# Supplementary material for: Are carbon emissions trading and green financial instruments synergistic? -Comprehensive quantitative research based on content analysis
Source: PLoS One. 2024 Mar 7;19(3):e0298601. doi: 10.1371/journal.pone.0298601 (PMC10919601; doi:10.1371/journal.pone.0298601)
Supplement: S1 Data — (ZIP) [file pone.0298601.s001.zip › Data/地级市Z_F工作报告.zip!/┴╔─■/┤≤┴1⁄4╩╨/└┤╫╘╣1⁄2╓┌║┼í╛┬φ┐╦╩2╛▌═°í┐_2016.docx]

各位代表：

　　现在，我代表市政府向大会报告工作，请予审议，并请市政协委员提出意见。

　　一、“十二五”时期和2015年政府工作回顾

　　“十二五”时期是大连发展极不平凡的五年。习近平总书记视察大连提出建设产业结构优化先导区和经济社会发展先行区的目标，为大连科学发展指明了方向；国务院批准设立金普新区，为大连实现更高水平改革开放提供了广阔舞台；国家确立大连新型城镇化综合试点，全域城市化全面推进。“十二五”时期也经受了国际金融危机的严重冲击，经历一系列大事、难事，遇到前所未有的严峻形势和经济下行压力。在省委、省政府和市委的正确领导下，市政府全面贯彻党的十八大和十八届三中、四中、五中全会精神，团结带领全市人民抢抓机遇、应对挑战、开拓进取、攻坚克难，经济社会发展取得了重大历史成就，谱写了振兴发展的新篇章，为率先全面建成小康社会奠定了坚实基础。

　　五年来，坚持把发展作为第一要务，综合经济实力显著增强。地区生产总值从5158亿元增加到7700亿元，年均增长8.5%.固定资产投资累计实现2.8万亿元，年均增长5.6%.公共财政收入累计达到3611亿元，年均增长2.9%.

　　五年来，坚持把调结构作为主攻方向，经济发展质量和效益持续提升。服务业增加值占GDP比重由42.7%提高到50.8%.金融业增加值占GDP比重由5.1%上升到9%,软件和信息服务业销售收入年均增长10%以上。传统制造业加快转型升级，战略性新兴产业异军突起，高新技术产品增加值达到720亿元，年均增长16.6%.

　　五年来，坚持把深化改革开放作为不竭动力，经济发展活力有效释放。行政审批、国资国企、投融资体制等多项改革顺利推进，营商环境进一步改善，民营企业营业收入、纳税总额分别增长38%和66%.五年累计利用外资536亿美元，完成出口总额1560.5亿美元，分别为“十一五”的2倍和1.5倍。境外协议投资年均增长50%.

　　五年来，坚持把全域城市化作为重大战略，城乡统筹发展协调推进。“四大组团”拓展了全域发展新空间，19个重点产业园区构建起多点支撑新格局。城市基础设施建设累计投资1831亿元，全域路网、电网、水网逐步完善。全市常住人口城镇化率五年提高10个百分点。

　　五年来，坚持把“三个中心”建设作为重要载体，城市功能日益完善。国际航运中心、物流中心和区域性金融中心建设取得新进展。全市港口货物、集装箱、空港旅客吞吐量五年分别增长31%、78.3%、28.5%.新增金融机构235家，新增社会融资超万亿元。

　　五年来，坚持把生态安全作为发展底线，环境质量不断改善。万元GDP综合能耗累计下降18%,单位工业增加值综合能耗年均降低4.6%.城市生活垃圾无害化处理率、生活污水处理率分别达到100%和95%,林木绿化率达到50%,海洋生态环境与资源保护取得新进展。

　　五年来，坚持把增进民生福祉作为根本宗旨，人民生活质量稳步提高。财政用于民生支出累计3050亿元，城乡居民收入年均分别增长10.4%和11.3%,人民群众得到了更多实惠。实现城镇实名制就业107万人，城乡社会救助、基本养老保险和医疗保险制度实现全覆盖。各项社会事业全面发展，文明城市建设成果不断巩固。

　　各位代表，2015年是“十二五”收官之年。在市委的正确领导下，市政府坚持稳中求进的工作总基调，主动适应和引领经济发展新常态，保持定力，沉着应对，认真落实习近平总书记提出的“四个着力”要求，坚持四化统筹、协调推进，全面落实稳增长、调结构、促改革、惠民生、防风险各项任务。预计地区生产总值增长4.2%.一般公共预算收入579.9亿元，下降25.7%.固定资产投资4600亿元，下降32.1%.全社会消费品零售总额3050亿元，增长8.5%.城乡居民收入达到35750元和14650元，分别增长6.5%和8%.城镇登记失业率2.93%.全市呈现经济平稳发展、民生持续改善、社会和谐稳定的良好局面。

　　各位代表，2015年难事多，大事、喜事也多。全市人民高度关注、对大连未来发展具有深远影响的一批重大政策、重大规划、重大项目，在多年不懈努力的基础上，集中力量，奋力攻坚，取得了重大成果：一是中国（大连）跨境电子商务综合试验区获得国务院批准；二是中日韩（大连）循环经济示范基地获得国务院批准；三是普兰店撤市建区获得国务院批准；四是大连历史上规模最大、投资740亿元的长兴岛恒力石化炼化一体化项目开工建设；五是我国利用外资单体规模最大、技术最先进的美国英特尔投资55亿美元的存储器项目落户我市；六是金州湾国际机场规划建设方案通过国家有关部门的初审；七是我市辖区渤海临岸国家级斑海豹保护区规划顺利通过国务院专家组的评审；八是开采近百年的后盐石灰石矿和营城子石灰石矿，经过与鞍钢集团多次协商，终于达成闭矿协议；九是投资270亿元的丹大快铁正式通车；十是总投资422亿元的地铁1号和2号线、星海湾大桥、莲花山隧道开通运营。这十件大事对进一步完善城市功能、提升城市品质、牵动未来发展、增强综合实力、增进民生福祉具有重大战略意义。

　　一年来，主要做了以下几个方面的工作：

　　（一）全力以赴稳增长，保持经济平稳健康运行。

　　多管齐下扩大有效投资。加大政府投资力度，完成公共基础设施投入119.4亿元。发挥财政资金杠杆作用，采取PPP模式推进14个示范项目建设，吸引社会资本参与202轨道延伸线、快轨3号线建设。全力推进重大产业项目开复工，促进优势产业投资不断增加。

　　多措并举促进消费增长。出台鼓励消费政策，激发旅游、信息、住房等十大领域消费需求，电商交易额实现3500亿元，大商集团销售额突破2000亿元大关，消费对经济增长贡献率达39%.加快房地产业结构调整，规划建设停车场81个、停车泊位2万个，大力推进老旧城区、棚户区改造，商品房销售面积和销售额逐步回升。接待国内外游客6200万人次，实现旅游收入1115亿元、增长12.5%.

　　多向发力稳定外贸出口。完善外贸鼓励政策，支持企业技术更新、产品翻新、商业模式创新，提升出口竞争力。强化船舶、服装、水产品等20个外贸转型示范基地建设，跨境电商交易实现38.4亿元。大力拓展外贸业务，出口市场扩展到196个国家和地区，对南亚、中东出口分别增长81.2%和37.5%.全市实现出口257.8亿美元，占全省的51%.

　　多方施策保障企业运行。出台25条优惠政策，对企业开拓市场、金融服务、技术改造等给予支持。加强企业运行监测，季分析、月调度，开展“走企业、解难题”活动，帮助企业解决重大问题75项。设立50亿元育龙基金，破解中小企业资金周转难题。支持企业债券融资2614亿元，比上年增长10.6倍。新增企业、注册资本分别增长9.6%和51.9%.为2.13万户小微企业减半征收所得税1.19亿元。

　　（二）加大力度调结构，不断提高经济质量和效益。

　　传统产业转型升级步伐加快。推动“两化”深度融合，促进传统产业提升竞争力。制定《中国制造2025》大连行动计划，船舶重工、大连中车、机床集团、华锐重工、光洋科技、瓦轴等一批装备制造业加快向系统集成和制造服务化转型。东风日产、奇瑞、黄海、华晨等生产整车19万辆，实现产值660亿元，汽车产业集群初步形成。船舶制造加速向高端产品转型，海洋工程产值达到150亿元，同比增长4.9%.

　　战略性新兴产业支撑能力增强。加大政策支持力度，促进一批特色鲜明的战略性新兴产业蓬勃发展。融科储能、比克电池、比亚迪新能源汽车等产业达产。中兴、华为等区域性研发中心、创新中心落户大连。战略性新兴产业业务收入达1300亿元，占规模以上工业产值16%.软件和信息技术服务主营业务收入达1630亿元。

　　现代服务业持续壮大。促进服务业与一二产业协调联动、跨界融合。金融业快速发展，增加值增长14%.采取筑巢引凤、腾笼换鸟等方式，推动PE大厦、信用产业园、忠旺新金融中心、融资租赁产业园等新兴业态加快发展，税收超亿元总部大厦达16座。发展冷链物流等省级服务业集聚区13个，主营业务收入增长8%.恒隆广场、柏威年、凯丹等商业综合体开门纳客。服务业增加值增长7%.我市成为国家电子商务与物流快递协同发展试点城市。

　　农业现代化稳步推进。战胜多年不遇的严重旱灾，农林牧渔增加值增长4%.落实国家政策，规范土地流转，创建都市型现代农业园区12个，新发展设施农业4万亩。7个投资10亿元以上的重大项目加快推进。旅顺口区成为全国休闲农业和海洋生态文明建设示范区，中国农科院都市农业研究院落户我市。

　　（三）突出重点抓改革，有效激发市场活力。

　　行政管理体制改革成效明显。创新金普新区行政体制，实现“大部制、扁平化”管理。全市取消、下放政府部门审批事项475项，率先公布市政府部门权责清单，职权项目精简2653项。市公共行政服务中心投入使用，34个政府部门699项行政审批和便民服务项目“三集中一分开”,实现一个窗口受理、一个印章审批、一个流程办结。实施“三证合一、一照一码”商事制度改革，推行建设项目“并联审批”.中国《公共服务蓝皮书》对全国各城市的公共服务评价中，我市民众满意度提升到第七位。

　　国资国企改革加快实施。启动市直党政机关所属企业和经营性事业单位改制，首批32户企业完成脱钩。建立健全国有资产管理和资本授权经营体制，组建国有资本投资运营公司，实现由管资产向管资本转变，国有企业净资产增长8.5%.完成大连金融资产交易所股权多元化改革。大连橡塑机与恒力集团实施资产重组。

　　医药卫生体制改革持续深化。实行医疗、医保、医药“三医联动”,县级公立医院综合改革深入推进，11所县级公立医院全部取消药品加价，全市181个基层医疗机构100%实施基本药物制度。社会资本兴办医疗机构累计达93所，城乡医疗卫生格局进一步优化。

　　（四）精准发力促开放，不断增强竞争优势。

　　开放型经济新体制逐步建立。出台新一轮对外开放若干意见，进一步加快与国际接轨步伐。金普新区在贸易、投资、口岸便利化等方面先行先试。口岸部门“三互”大通关和国际贸易“单一窗口”建设走在全国前列。复制上海自贸区11项海关监管制度，实施5项检验检疫便利措施。保税区、高新区、长兴岛、花园口等对外开放先导区配置资源要素的能力和效率进一步提升，集聚产业功能明显增强。

　　招商引资成效显著。把招商引资作为稳增长、调结构的重要抓手，先后组织18个团组赴16个国家（地区）和北京、上海、深圳等城市，开展登门招商、产业链招商、外商内招，引进内资1805亿元、亿元以上项目86个。新批外资项目203个、利用外商直接投资27亿美元，占全省的53%.引进固特异中国研发中心、IBM中国创新中心、美国CDK软件研发中心、日本TDK财务共享中心。实践表明，大连仍然是吸引外资具有竞争力的城市。

　　“走出去”实现新突破。抢抓“一带一路”战略机遇，企业“走出去”和国际产能合作成效明显。大机床美国研发中心、瓦轴美国检测中心和欧洲研发中心挂牌运营，远东工具项目落户瑞士。万达集团、三寰集团等企业跨国发展势头强劲。全市新批境外投资项目108个，中方协议投资33.6亿元、增长17.5%.

　　国际性会展成果丰硕。夏季达沃斯论坛影响力进一步扩大，坚持以会交友、以会招商，促成合作项目13个、投资额达81.4亿美元。“软交会”溢出效应明显，30家世界500强企业参展，签约信息产业项目15个。“服博会”参展国际品牌454个，创新“互联网+展会”模式，使“服博会”成为网上“永不落幕的服装节”.国际车展吸引12个国家1300辆新款汽车参展，啤酒节接待中外游客150万人次。

　　（五）创新驱动添动能，加速形成发展新引擎。

　　创新体系加快完善。落实国家促进创新创业政策，出台配套措施27项。打造60个研发与产业化贯通的创新平台，辽宁精细化工共性技术创新平台落户我市。推进区域性科技创新创业中心建设，与中科院39家院所和34所“985”大学建立成果转化机制，高新区成为全国科技服务创新试点区。

　　自主创新能力显著提升。发挥科技创新主导作用，构建企业为主体、产业为纽带、金融为支撑的10个产业技术创新联盟。新增国家级高新技术企业119家，技术先进型服务企业123家，并购海外科技型企业42家。中小企业开发“专精特新”产品76项。每万人发明专利拥有量11.5件，技术交易额增长16%.在刚刚闭幕的国家科技奖励大会上，我市10个项目获得奖励。

　　大众创业、万众创新蓬勃兴起。成立市创业促进委员会，指导、协调和推进创新创业活动开展。探索“泛在式”科技创业模式，科技企业孵化器达到39家、众创空间29家。高新区创造的“互联网+众创”模式，受到李克强总理的称赞。金普新区高技能人才实训基地和创业孵化基地投入使用，北大创业训练营落户大连，全年实现创业就业3.5万人。

　　（六）注重内涵强功能，进一步提升城市品质。

　　“三个中心”建设深入推进。大连港加速向第五代港口转型升级，太平湾港区规划通过省部评审。打造国际陆海联运大通道，港口与“一带一路”重要节点城市实现高效联动，物流业增加值占GDP比重达10.6%.区域性金融中心建设步伐加快，全球金融中心指数位列国内城市第4位。新增各类金融机构47家，新增上市挂牌企业39家。大连银行成功引进战略投资150亿元，资产质量明显提升。大连商品交易所交易规模再创新高，成交量、成交额分别占全国商品期货市场的34%和30.6%.

　　统筹城乡发展力度加大。全域基础设施一体化建设步伐加快，渤海大道、金普城际铁路、河口交通枢纽等建设工程加快推进。高起点实施新型城镇化综合试点，皮杨等11个中心镇实施“强镇扩权”.建立农业转移人口成本分担机制，市政府投资17.8亿元，支持新型城镇化重点区域基础设施和公共服务项目建设。深化户籍制度改革，实行居住证制度，推进农业转移人口市民化，全市常住人口城镇化率提高到75%.

　　智慧城市建设稳步推进。制定并实施智慧城市规划，整合资源，理顺机制，有序推进智慧城市建设。“宽带大连”实现城区光纤化，4G信号实现城区全覆盖，用户达287万个。智慧社区、智慧街道、智慧旅游、智慧交通、智慧卫生等便民工程加快推进。

　　城市文化内涵不断提升。现代公共文化服务体系和文化创新环境进一步完善，培育形成一批文化品牌。旅顺太阳沟文化产业园区等重点文化产业项目加快推进，促进文化与科技、旅游等产业融合发展。公益演出400场，建设农村“文化大院”500个。我市第四次被评为全国文明城市。

　　城市环境整治成效显著。深化城市管理体制改革，推动行政执法重心下移。市区联动，综合执法、公安、城建、民政、工商、卫生等部门密切配合，大力整治占道经营、违章建设、无证营运、街巷烧纸等影响市容环境的突出问题，广大市民坚决拥护、主动参与，共同打赢了一场环境综合整治攻坚战。全市共取缔无证营运机动车8.2万辆，拆除违章建筑27万平方米，清除沿街堆放杂物6000多车，香炉礁马路市场等多个群众反映强烈的环境顽疾被清除。全年植树6100万株，造林补植28.2万亩。我市获得全国生态文明示范区称号。

　　（七）千方百计惠民生，社会事业全面进步。

　　民生投入不断加大。全年用于民生方面的支出667.9亿元，占财政总支出的73.3%,高质量完成15项重点民生工程，实现了政府对人民的庄严承诺。加快保障性安居工程建设，完成棚户区改造1.24万套、“暖房子”工程300.7万平方米；实施城市建设“五个一”工程127项、住房维修“一二三”工程628项。新建通屯油路800公里，新建农村安全饮水工程18项，解决了1.55万人饮水安全问题。

　　社保能力得到增强。持续提高社会保障水平，企业退休人员基本养老金提高到每月2258元，城乡居民基础养老金提高到每月195元，城镇居民医保、新农合财政补助标准达到480元，城乡低保标准每人每月分别达到610元和380元。启动机关事业单位养老保险制度改革。加大政府促进就业力度，完善就业服务体系，实现城镇实名制就业22.8万人。

　　社会事业全面发展。坚持普惠性、均等化、可持续原则，不断提升社会事业服务能力和共享水平。新开办普惠性幼儿园21所，长兴岛、花园口和长海县通过国家义务教育均衡发展验收。优化职业教育专业设置，对16所院校的36个专业进行整合。高校人才培养、科技创新、文化传播能力不断增强，对城市发展的作用进一步凸显。市规划展示中心等一批重大工程竣工，大连市烈士陵园落成。名医、名科、名院建设稳步推进，中医药事业长足发展，医疗软实力日益增强。全民健身运动广泛开展，完成城市健身场地51处，市民健身中心投入使用。废弃多年的火车头体育场改造工程开工。40所学校成为全国“校园足球”特色校，重组后的大连一方足球俱乐部赛绩明显提升。市第十二届运动会圆满成功。

　　社会治理不断加强。针对安全生产的严峻形势，制定安全生产攻坚战方案，以强化安全意识、健全安全制度、推行安全标准为重点循序推进安全生产工作，以超常力度、超常举措扭转了安全生产的被动局面。年初分层次召开所有法人单位负责人参加的动员大会，组织7.8万个单位法定代表人约谈对话，增强全社会成员的安全意识。开展隐患排查百日专项治理和“百名记者百日行”活动，全市工矿商贸企业事故起数、死亡人数分别下降51%和49%,较大以上事故发生起数、死亡人数分别下降75%和73%,企业本质安全水平大幅提升。开展食品药品监管“五个一”集中行动，实施16项监管工程，食品药品安全水平进一步提高。社区建设不断加强，社会组织参与社会治理达95%以上，信访总量下降14.3%.社会治安立体化防控体系建设成效显著，工作经验在“大连会议”上向全国推广。

　　（八）真抓实干转政风，深入推进依法行政。

　　法治政府建设加快推进。全面贯彻党的十八届四中全会精神，自觉在法治轨道开展工作。创新执法体制，完善执法程序，推进综合执法，严格执法责任，建立了权责统一、权威高效的行政执法体制。依法履行政府职能，坚持法无授权不可为、法定职责必须为，建立起行政机关敢于担当、敢于负责的激励评价机制。

　　决策程序进一步完善。把公众参与、专家论证、风险评估、合法性审查、集体讨论决定作为重大行政决策的法定程序。坚持重大事项决策前向人大报告、与政协民主协商，认真落实人大执法检查、视察、专题询问和政协协商意见。积极回应人大代表、政协委员对社会热点问题的关切，609件人大代表建议、707件政协提案全部办复。自觉接受人大法律监督、工作监督和政协民主监督，认真听取各民主党派、工商联、无党派人士和人民团体意见。全面推行政务公开，让权力在阳光下运行。

　　行政效能不断提高。扎实开展“三严三实”专题教育，政府各部门和广大公务员主动适应经济发展新常态，以严和实的精神，推动各项工作落实。各级领导干部勇于负责、勤奋敬业，越来越多的部门和工作人员把干事、成事作为一种追求，政府的执行力和工作效率进一步提高。在履行职责、服务群众中，广大干部不辞辛劳、努力工作，有6位同志因公殉职，涌现出孙德忠等一批忠于职守、无私奉献的先进典型。

　　廉洁从政措施全面落实。认真践行政府系统廉洁从政“五项承诺”,加大对不作为、不担当、乱作为和失职、渎职等问题的追究和问责力度，立案调查145件，党纪政纪处理150人。切实加强政风行风建设，坚决查处发生在群众身边的“四风”和腐败问题，保持了惩治腐败的高压态势。强化行政监察和经济责任审计，严控“三公”经费支出，全年“三公”经费同比下降15%.

　　民族宗教、残疾人、外事、侨务、对台、人防、档案、气象、防震减灾、仲裁、双拥共建、民兵预备役、边海防等部门恪尽职守、扎实工作，都取得了新成绩。

　　各位代表，五年的成绩来之不易！凝结了社会各界的心血、智慧和汗水，全市人民付出了艰辛的努力。在此，向给予政府工作大力支持的人大代表和政协委员，向各民主党派、工商联和无党派人士，向离退休老同志，向中省直各部门和兄弟省市驻连机构，向驻连部队和武警官兵，向关心支持大连发展的海内外朋友们表示衷心的感谢！

　　在肯定成绩的同时，我们也要清醒地看到存在的问题：一是体制机制依然不活，改革的系统性、整体性、协同性有待加强；二是部分经济指标下降幅度较大，有效投资不足，出口持续下滑；三是工业产能利用率降低，经营成本增加、效益下降；四是经济结构调整任重道远，去产能任务繁重；五是企业创新能力不强，新的增长动能还没有形成支撑力量；六是财政收入下降与民生刚性支出增长矛盾突出；七是城市内涵品质亟待提升，交通拥堵、空气污染较重；八是有的干部缺乏工作激情和改革创新锐气，在困难面前不敢作为、不愿担当，政府自身建设还需进一步加强。这些问题必须引起高度重视，在今后工作中切实加以解决。

　　二、“十三五”时期发展思路和目标任务

　　未来五年是我市率先全面建成小康社会的决胜阶段，是推进结构性改革的关键时期。我们要以党的十八大和十八届三中、四中、五中全会精神为指导，深入贯彻习近平总书记系列重要讲话精神，按照“五位一体”总体布局和“四个全面”战略布局，牢固树立和贯彻落实创新、协调、绿色、开放、共享的发展理念，坚持发展是第一要务，以提高经济质量和效益为中心，加快形成引领经济发展新常态的体制机制和发展方式，抓住机遇、保持定力，着力完善体制机制，着力推进结构调整，着力鼓励创新创业，着力保障和改善民生，坚持稳中求进，四化统筹、协调推进，加快“四个中心、一个聚集区”建设，为实现产业结构优化的先导区和经济社会发展的先行区目标而努力奋斗！

　　“十三五”时期的主要目标是：经济实力大幅跃升。经济保持中高速增长，在提高发展的平衡性、包容性和可持续性的基础上，地区生产总值比2010年翻一番，迈入万亿城市行列。人民生活水平普遍提高。城乡居民人均收入比2010年翻一番以上，市民文明素质和社会文明程度显著提高。经济结构持续优化。服务业占比进一步提升，传统产业转型升级步伐加快，战略性新兴产业对经济的支撑作用明显增强。城市功能进一步完善。核心城市地位进一步巩固，对要素的吸纳能力和经济辐射能力明显提升。生态环境质量总体改善。万元GDP综合能耗继续降低，污染物排放持续下降，自然生态得到修复，林木覆盖率进一步提高，大气质量明显改善。

　　“十三五”时期经济社会发展要突出“五个坚持”:

　　坚持把增进民生福祉作为一切工作的出发点和落脚点。不断实现好、维护好、发展好最广大人民的根本利益。完善收入分配格局和调节机制，千方百计增加城乡居民收入。推进基本公共服务均等化，进一步提高公共服务共建能力和共享水平，使全市人民更广泛参与发展过程、更公平分享发展成果。

　　坚持把创新驱动作为引领发展的主导战略。把创新摆在发展全局的核心位置，以科技创新引领全面创新。着眼推动产业转型升级、发展动力转换，加快构建创新的体制机制，围绕产业链布局创新链，培植创新创业文化，激发创新创业活力，加快推进科技与经济深度融合。

　　坚持把深化改革开放作为推动发展的根本动力。发挥对外开放优势，融入全球开放合作，主动对接“一带一路”和京津冀协同发展战略。坚持“引进来”和“走出去”并重，引资、引智和引技并举，用好国际国内两个市场、两种资源，构建开放型经济新体制。全面深化改革，坚持市场驱动，充分发挥市场在资源配置中的决定性作用和更好发挥政府作用。

　　坚持把绿色发展贯穿于经济社会发展全过程。坚定走生产发展、生活富裕、生态良好的文明之路，建设资源节约型、环境友好型社会，倡导绿色循环低碳生产方式、生活方式，建设山青、水绿、天蓝的美丽大连。

　　坚持把依法治市作为推进治理体系和治理能力现代化的重要载体。运用法治思维和法治方式推动发展。把政府管理和经济社会发展纳入法治轨道，着力统筹社会力量、平衡社会利益、调节社会关系、规范社会行为，强化法治的引领、规范和保障作用，创新社会治理，以行政权力的“减法”换取市场活力的“乘法”.

　　各位代表，大连“十三五”规划纲要草案已提请大会审议，历史使命光荣，奋斗目标宏伟，我们必须只争朝夕、奋发有为。

　　当前，世界经济仍处在深度调整和缓慢复苏之中，不确定、不稳定因素增加，形势不容乐观。国内经济发展进入新常态，经济增速换挡、结构调整阵痛、动能转换困难相互交织，有效需求乏力和有效供给不足并存，下行压力仍然很大。与此同时，我们也应看到重大发展机遇正在转换：由原来加快发展速度的机遇转变为加快发展方式转变的机遇，由原来规模快速扩张的机遇转变为提高发展质量和效益的机遇。综合分析判断：我市经济发展长期向好的基本面没有变，经济持续增长的支撑基础和条件没有变，经济结构调整优化的前进态势没有变。新兴产业迅速发展，新的增长点正加快孕育并不断破茧而出，社会创新能力不断增强，新的增长动力正加快形成并不断积蓄能量。我市拥有雄厚的物质基础和要素资源，改革开放将不断释放巨大红利。面对未来，我们充满信心！

　　三、2016年主要工作

　　2016年是“十三五”的开局之年，对于高质量完成“十三五”任务至关重要。我们必须认识新常态、适应新常态、引领新常态，牢牢把握经济发展大逻辑，认真落实“十三五”规划的总要求，围绕奋斗目标和重点任务，加强供给侧结构性改革，去产能、去库存、去杠杆、降成本、补短板，努力实现“十三五”良好开局。主要预期目标是：地区生产总值增长6.5%-7%,固定资产投资增长6%左右，社会消费品零售总额增长10%左右，外贸出口增长6%左右，一般公共预算收入、城乡居民人均收入与经济发展同步。万元GDP综合能耗下降3.5%,CPI控制在3%以内，城镇登记失业率控制在3%以内。

　　实现上述目标，要着力做好以下工作：

　　（一）着力推动经济平稳增长。把稳增长放在更加突出位置，坚持供给侧与需求侧同时发力，推动经济持续健康发展。

　　努力保障实体经济健康运行。全力为企业服务，改善企业市场预期，全面落实国家和省市支持企业发展政策，在减少干扰、保障要素供应等方面打出“组合拳”.深入实施“育龙计划”,着力化解民营企业融资难、准入难、创新难等突出问题，营造更加公平公正的市场环境，让民营企业成为经济发展生力军。

　　持续扩大有效投资。突出投资的有效性和精准性，注重强功能、调结构、补短板、惠民生，实施一批新的重点项目建设，增加公共产品有效供给。全年实施亿元以上项目630个，总投资1.5万亿元。加强基础设施建设，实现渤海大道一 期全线贯通，完成地铁1号和2号线二期、金普城际铁路主体工程建设，加快推进旅顺中部通道、疏港路拓宽改造工程，推进金州湾国际机场、长海机场建设，开工建设地铁4号、5号、7号线和大连湾跨海交通、大连湾海底隧道等重大工程。完成金普城际铁路延至瓦房店的前期工作。积极化解房地产库存，培育房屋租赁市场，加快旧城区和棚户区改造。大力调整房地产结构，引导房地产业向健康地产、养老地产转型。加快停车场规划和建设，新增停车泊位30万个。加快重大产业项目开复工，推动英特尔存储器、中石油炼化、固特异三期等270个重大产业项目建设。运用股权、基金、债券等手段拓展融资渠道，采取PPP等方式推进项目建设，有效释放社会投资潜力。

　　积极发挥新消费引领作用。把握消费由传统型向个性化、时尚化发展的新趋势，实施服务、信息、绿色、时尚、品质、农村6大领域消费工程，促进旅游消费、健康消费、住房和汽车等大宗消费。扩大信息消费，推进电信普遍服务。大力发展电子商务、智能家居等服务产业。推行使用权短期租赁等分享经济模式，进一步增加中高端消费。办好软交会、服博会、啤酒节等活动，促进节庆会展消费。

　　加快培育外贸竞争新优势。坚持增加总量与提升质量并重，推动对外贸易优进优出。促进外贸结构调整，鼓励绿色低碳和战略性新兴产业扩大出口。抓好出口基地建设，开展“大连产品”走出去行动，精心培育出口品牌。巩固传统市场，大力开拓新兴市场。发挥外贸专项资金作用，引导出口企业更新产品、抵御风险。创新外贸发展模式，加快发展采购贸易、服务贸易、技术贸易。

　　（二）着力优化产业结构。做好“加减乘除”大文章，注重培育壮大“六大新的增长点”,走质量型、内涵式发展道路。

　　促进传统产业转型升级。实施《中国制造2025》大连行动计划，加快推进创新中心建设、质量品牌提升、高端装备创新以及工业强基、智能创造、绿色制造六大工程建设。做大以东风日产、奇瑞、比亚迪和华晨等整车生产企业为牵动的汽车及零部件产业集群，完善产业链。大力发展绿色石油化工，加快恒力石化炼化一体化、催化产业之都等重大项目建设。推动服装、家具、食品等产业技术、品牌、价值升级。加快军民产业融合示范基地建设。推动制造业向智能化、网络化、服务化和价值链高端延伸，为传统产业插上腾飞的“翅膀”.

　　支持战略性新兴产业快速成长。大力发展集成电路、储能装备产业，推动软件和信息技术服务、新能源、新材料、3D打印、生物医药、海洋新兴产业快速发展，创建国家级新能源汽车产业示范基地。加快发展新一代信息技术，推进移动互联网、大数据、云计算、物联网与制造业融合发展。抓好国家电子商务与物流快递协同发展试点城市和电子商务示范县建设，实施电子商务应用推进工程。

　　推动服务业向规模化高端化发展。强化国际航运中心功能，提升核心港区服务能力，培育东北亚国际航运交易市场，完善物流中心集疏运体系。提升保税港区功能，扩大保税仓储、国际物流配送等业务，做大做强物流产业集群。全面实施《大连区域性金融中心建设促进条例》，推进金融服务八项重点工程，全年新增金融机构20家、上市挂牌企业30家，支持大商所加快建设多元开放的综合衍生品交易中心，改善金融生态环境。推动国际贸易中心建设，打造具有区域影响力的商品集散、冷链物流、国际采购和展示交易中心。大力发展“平台经济”,支持大连再生资源交易所等平台发展。加快东港、梭鱼湾、机场商务区建设。深度开发海岛、温泉、休闲等旅游精品，打造东北亚旅游胜地。

　　大力发展都市型现代农业。积极推进农业产业化，发展多种形式适度规模经营，促进特色农业向优势区域集聚，新增设施农业5万亩，创建都市型现代农业园区15个，加快建设海洋牧场。大力发展绿色、循环、特色和品牌农业，创新金融支农惠农服务机制，创新农产品流通方式，为农民生产生活提供更加有效的服务。完成土地承包经营权确权登记颁证300万亩。

　　（三）着力鼓励创新创业。把创新作为第一动力，把人才作为第一资源，加快新动能成长和传统动能提升。

　　完善“产学研用”相结合的创新体系。进一步引导企业加大技术创新投入，设立装备制造业创新引导基金，搭建检验检测公共服务平台。建立技术创新战略联盟，强化高校和科研院所对企业技术创新的源头支持，促进产学研用协同创新，运用市场机制促进科技成果本地转化。支持金普新区、高新区创建国家自主创新示范区，建设区域性创新创业中心。

　　推进创新链与产业链有机融合。强化企业创新主体地位，开展关键共性技术研发攻关，支持企业在核心技术上实现突破，形成一批具有自主知识产权的技术和产品。围绕产业链部署创新链、完善资金链、整合人才链、提升价值链，加快培育壮大一批创新型龙头企业。

　　全力支持大众创业、万众创新。发挥创业促进委员会及常设工作机构的作用，指导和推动群众性创业活动深入开展。大力发展众创、众包、众扶、众筹，发挥“双创”集众智汇众力的乘数效应，加快建设综合性创新创业产业园。以创新促进新产品、新技术、新业态、新模式发展，培育新的增长点。创新天使投资、创业投资、风险投资等金融支持模式，引导社会资本积极参与创新创业。实施高层次人才创新支持计划、创业培训“千人计划”、大学生创业引领计划，发挥“海创周”引才纳智平台作用，在全社会营造靠创业自立、凭创新出彩的浓厚氛围。

　　（四）着力深化各项改革。破解发展难题，必须深化改革，激发市场活力和社会创造力。要围绕解决重点领域的突出矛盾和问题，抓住牵一发而动全身的关键环节推进改革。

　　深化简政放权、放管结合、优化服务改革。聚焦解决束缚大众创业、万众创新和企业投资经营、便民服务的突出问题，把“放、管、服”引向深入，转变政府职能，提高行政效能，防止权力寻租，铲除滋生腐败的土壤。进一步深化商事制度改革，巩固扩大“三证合一、一照一码”改革成果。进一步增强改革的协同性，防止“中梗阻”,打通“最后一公里”.进一步提高监管的有效性，全面推开“两随机、一公开”,有效解决检查任性、执法不公和执法不严等问题，创新监管方式，推进综合执法和大数据监管，运用市场、信用、法治等手段协同监管。进一步强化行政服务的便利性，推出告知承诺制等服务新举措，加快政府审批标准化建设，切实解决“办证多”“办事难”等问题。

　　深化财税和投融资体制改革。认真贯彻新《预算法》，实行规范透明预算制度，除涉密信息外都要公开预决算。全面实施“营改增”,及时调整财政收入结构安排。创新财政支出方式，发挥政府投资“四两拨千斤”的作用，提高财政资金使用效率和带动效应，在基础设施和公用事业领域引导社会资本参与重点项目建设，增强政府再投资和公共产品供给能力。健全金融为实体经济服务的体制机制，加快发展服务小微企业的中小金融机构。落实地方政府金融监管责任，完善监管体系，防范和化解金融风险。

　　打好国有企业改革攻坚战。完善国有资产管理体制，从管资产为主转向管资本为主。优化国有企业结构布局，加快从非主业领域、缺乏竞争优势的领域及一般产业的低端环节退出，严控产能过剩行业投资，促进国有资本更多向前瞻性、战略性产业和一般产业的中高端集中，培育市场竞争新优势。赋予国有企业改革自主权，鼓励国有企业混合所有制改革，运用市场机制促进产业兼并重组，完成党政机关所属企业和经营性事业单位脱钩改制任务。

　　（五）着力扩大对外开放。加快实施新一轮对外开放，构建开放型经济发展新体制。

　　完善对外开放新格局。加快金普新区开发开放，在金融服务、土地管理、开放型经济、商事制度、通关便利化等方面先行先试，打造面向全球开放合作的战略高地。全力申办自由贸易试验区，积极复制上海自贸区的经验，支持口岸部门“大通关”改革，推进投资、贸易便利化。加速金融业开放，扩大人民币跨境使用，开展外汇管理创新试点。积极推进中国（大连）跨境电子商务综合试验区规划建设。加快中日韩（大连）循环经济示范基地建设。鼓励各开放先导区创新体制机制和开发模式，形成新的资本、人才、技术集聚效应。

　　拓展国际合作新空间。积极对接“一带一路”战略，发挥政府政策支持、项目发布、信息咨询和风险预警平台作用，大力推进国际产能合作，支持企业承揽境外工程项目。鼓励建设“海外仓”和展示中心。引导企业通过资本扩张、海外并购等方式，增强要素融合、市场共享、技术提升和产品创新能力，提升国际竞争力。

　　掀起招商引资新热潮。强化全员参与，以更大的力度推进招商工作。坚持内外资并重，引资与引智、引技结合，提高引资规模和质量，加快引进和储备一批重大产业项目，充分利用存量资本吸引国内外增量资本，鼓励企业境外上市、融资租赁，吸引跨国公司在连设立区域性总部和研发中心。实现直接利用外资超过30亿美元、引进内资超过2000亿元。

　　（六）着力发展民生社会事业。不断加大民生投入，切实保障和改善民生，让人民群众拥有更多获得感。

　　扎实开展重点民生工程建设。千方百计提高城乡居民收入，改善收入结构，努力提高经营性和财产性收入比重。坚持保基本、补短板、提标准，高质量实施18项重点民生工程。加大财政民生支出力度，确保用于民生支出继续增加。

　　强化就业和社会保障。加大政策扶持力度，重点做好高校毕业生、下岗失业人员、农村转移劳动力和退役军人就业，新增城镇就业8万人。进一步完善社保制度，提高城镇职工基本养老金待遇水平和城乡居民医疗保险财政补助标准，实现城乡居民养老保险、医疗保险全覆盖。健全以居家养老为基础、社区养老为依托、机构养老为补充、医养结合的养老服务体系。坚持精准扶贫，大力解决农村贫困群众的生活困难。推进保障性安居工程建设，实施棚户区改造8071套，新增公租房租赁补贴5000户。推进城市建设“五个一”和住房维修“一二三”工程，完成“暖房子”工程300万平方米。

　　让市民享有更优质的教育。促进义务教育均衡发展，支持普兰店、瓦房店、庄河创建国家义务教育均衡县。推动普通高中多样化发展，加强教师培训，促进民办教育质量提升。加快职教基地及配套设施建设，推动职业教育集团化办学。继续推进公办幼儿园建设，扶持民办幼儿园“降价提质”普惠发展。全面实施乡村教师支持计划，帮助农村青年教师解决生活困难。加大对在连高校创新发展的支持力度。

　　加快提升医疗卫生服务水平。以建设“健康大连”为目标，深化医药卫生体制改革，继续实施“三医联动”“三名工程”,优化医疗机构布局，鼓励发展民营医疗机构，推动中医药事业发展，持续提升医疗技术和服务水平，增强重大疾病预防控制能力。加快人口健康信息化建设，积极落实国家人口政策。

　　继续做好民族宗教、残疾人、外事、侨务、对台、人防、档案、气象、防震减灾、仲裁、双拥共建、民兵预备役和边海防等各项工作。

　　（七）着力提升城市功能和品质。坚持品质立市、建管并重，优化城市布局，促进生产空间集约高效、生活空间宜居适度、生态空间山清水秀。

　　提高城市发展宜居性。尊重、顺应城市发展规律，按照“五个统筹”的要求，加强城市的空间立体性、平面协调性、风貌整体性、文脉延续性的规划和管控。提高城市管理和服务水平，落实城市管理主体责任，实现城管执法重心下移，将城市环境综合整治进行到底，不断巩固和扩大整治成果。坚持“软引导”和“硬约束”并重，采取有力措施治理城区乱停车、乱占道等行为，妥善解决交通拥堵、“马路拉链”等人民群众反映强烈的问题。完善城区地下管网管理制度，大力推进城市地下综合管廊和“海绵城市”建设。深入开展城乡结合部和农村环境连片整治，全面优化城乡环境。

　　深入推进全域城市化。实施“多规合一”、统筹发展，加快推进全域性重大基础设施建设，提高城乡公共服务均等化水平。坚持产城融合发展，支持园区承接产业转移，实现以“业”兴“城”,稳步推进农业转移人口市民化。建立健全“人、地、钱”三挂钩机制，推动卫星城和中心镇协调发展。深入实施新农村“六化”工程，加快建设美丽乡村。

　　大力推进智慧城市建设。以国家信息消费示范城市建设为载体，突出“为民惠民”宗旨，在口岸、交通、教育、文化、医疗、旅游、公共管理等领域推出一批“互联网+城市”示范工程。通过政府引导、社会资本参与，加快“宽带大连”“无线城市”建设，实现信息资源共享、城市运转高效、公共服务便捷，打造东北亚重要信息港。

　　加快建设文化强市。大力弘扬时代精神和城市文化，积极培育和践行社会主义核心价值观，提升市民文明素养。推进历史博物馆、普湾图书馆、科技馆等项目建设，年内要竣工投入使用。加强太阳沟等历史文化资源保护。培养壮大作家、艺术家队伍，鼓励创作更多艺术精品。推动群众性文化活动开展，组织公益演出400场。加快文化产业发展，推动文化与科技、旅游等有机结合，促进文化创意、设计服务等业态发展。广泛开展全民健身运动，推动体育产业加快发展，振兴足球事业。

　　大力实施生态文明建设。着力推进国家生态文明示范区建设，促进绿色低碳循环发展。深入实施大气、水污染防治行动计划，强化总量控制，推进污染减排。实施煤改电、淘汰黄标车、划定高污染燃料禁燃区等十项举措，加强雾霾治理。从今年起，新增公共用车全部实现电动化。强化海洋环境监测，实施海域、海岛、海岸生态整治修复工程。搞好废弃矿区环境治理，高标准建设一批山体公园，让好山好水好风光融入城市。

　　（八）着力加强和创新社会治理。巩固社会治安防控体系建设成果，大力构筑共建共享的社会治理格局。

　　夯实社会治理基础。完善市民综合服务、矛盾纠纷化解、社会组织孵化和“平安大连”建设四个工作体系，实现上下贯通、全面覆盖和有效运行。强化社区服务功能，引导社会组织参与社会管理和公共服务，建设社区、社工、社会组织联动工作机制，增强基层社会自治能力。

　　健全公共安全体系。筑牢安全生产防线，全面落实“党政同责、一岗双责、失职追责”制度和企业全员安全生产责任制。开展企业安全生产管理体系认证，建立常态化、规范化隐患排查机制，提升企业本质安全水平。加大监管执法力度，严格事故查处和责任追究。创建国家食品安全示范城市，健全从生产到消费的全过程食品安全追溯体系。加强危机预防和应急管理，提高对自然灾害、事故灾难、公共卫生和社会安全事件的应急处置能力。

　　做好社会稳定工作。坚持法定途径优先原则，统筹发挥诉讼、仲裁、行政和人民调解作用，健全畅通有序的诉求表达、心理干预、矛盾调处、权益保障机制。强化信访工作责任落实，及时就地解决群众合理诉求。加大投入和创新力度，提升公共安全领域科技水平，增强信息化条件下驾驭社会治安局势的能力。

　　（九）着力推进政府自身建设。加快建设法治政府、创新政府、服务政府和廉洁政府，不断增强执行力和公信力。

　　坚持全面依法行政。加快法治政府建设，深入推进依法治市。完善政府依法决策机制，严格履行重大行政决策法定程序。自觉接受人大及其常委会的法律监督和工作监督、政协的民主监督、公众和舆论监督，自觉在法治轨道上开展工作。

　　持续改进工作作风。牢固树立群众观点，一事当前，都要听一听人民的心声诉求，算一算群众的利益得失，问一问百姓的意见建议，真心实意地为基层办事、为群众服务。认真落实“三严三实”要求，大兴主动作为、勇于担当、勤勉敬业、敢做善成之风，认真解决少数干部不作为、乱作为、不担当等突出问题。以踏石留印、抓铁有痕和“马上就办”的作风，狠抓工作落实。要保持一股“闯劲”、一股“钻劲”、一股“拼劲”,唯“闯”才有新路，唯“钻”才有新招，唯“拼”才有新得。要一项任务一项任务完成，一个项目一个项目落地，一个节点一个节点推进，增强执行力、提高战斗力。全面推进政务公开，建设阳光政府。

　　强化反腐倡廉建设。严格执行党风廉政建设责任制，自觉遵守廉洁从政各项规定，强化对政府部门履职情况的监督，建立健全行政问责追究机制。严肃查处发生在重点领域、关键环节和群众身边的腐败案件，坚决整治侵害群众利益的不正之风，树立风清气正、勤政为民的良好形象。

　　各位代表，时代赋予重任，拼搏铸就辉煌。让我们紧密团结在以习近平同志为核心的党中央周围，在市委的坚强领导下，心系百姓勇于担当、勤勉尽责奋发有为，以更加饱满的激情、更加务实的作风，同心同德，开拓创新，扎实工作，砥砺奋进，为实现全市人民的美好梦想而不懈奋斗！
